# Increased CD160 expression on circulating natural killer cells in atherogenesis

**DOI:** 10.1186/s12967-015-0564-3

**Published:** 2015-06-13

**Authors:** Jin Zuo, Zhaoliang Shan, Lin Zhou, Jian Yu, Xiaopeng Liu, Yuan Gao

**Affiliations:** Department of Cardiology, Navy General Hospital of Chinese PLA, Beijing, 100863 China; Department of Cardiology, General Hospital of Chinese PLA, Beijing, 100853 China; Department of Interventional Radiology, 302 Hospital of Chinese PLA, Beijing, 100039 China; Center of Health Examination, Navy General Hospital of Chinese PLA, Beijing, 100048 China

**Keywords:** Atherosclerosis, CD160, NK cells

## Abstract

**Background:**

Atherosclerosis (AS) presents characteristic of a chronic inflammatory disease in which both adaptive and innate immune cells play roles. Accumulating evidence has showed the impairment of natural killer (NK) cells in atherosclerosis, however, the mechanisms of this impairment remain unclear. In this study, we investigated the expression of CD160 on NK cells and assessed its pathological roles in NK loss during atherogenesis.

**Methods:**

CD160 expression on NK cells was measured in 49 AS patients and 41 healthy controls (HC) by flow cytometry, their inflammatory cytokine levels in sera were determined by ELSIA, and the effect of CD160 engagement on NK cells was evaluated by in vitro culture experiments.

**Results:**

Compared to HC, AS patients had a significantly increased CD160 expression on peripheral NK cells and concomitantly decreased peripheral NK cell number, and increased CD160 expression was positively related to the levels of serum lipids and IFN-γ, TNF-α and IL-6 inflammation cytokines, which all are risk factors for atherogenesis, and inversely correlated with peripheral NK cell number. Furthermore, engagement of CD160 receptor on NK cells from AS patients triggers a significantly increased production of inflammation cytokines and subsequent NK cell apoptosis, and blockade of TNF-α prevented the increased apoptosis of NK cells from AS patients after CD160 engagement, indicating a critical role of TNF-α in mediating NK cell loss by CD160 engagement.

**Results:**

Our results provide evidence that elevated CD160 expression on NK cells plays an important role in NK cell loss in atherosclerosis. The increased CD160 expression on NK cells might be used as an indicator for disease progression.

**Electronic supplementary material:**

The online version of this article (doi:10.1186/s12967-015-0564-3) contains supplementary material, which is available to authorized users.

## Background

Atherosclerosis (AS) is a chronic inflammatory disease of elastic and large muscular arteries, characterized by lesions containing cholesterol, immune cells, smooth muscle cells, and necrotic cores [1]. A key component of atherosclerotic plaque inflammation is the presence of different innate immune cell types including mast cells, neutrophils, natural killer cells, monocytes, macrophages and dendritic cells [2, 3]. NK cells are bone marrow-derived innate immune cells that sense pathological changes in tissues through the balanced cognate activities of inhibitory and activating receptors, which respectively recognize the reduced expression of major histocompatibility complex (MHC) class I molecules and the increased expression of MHC class I homologues such as MICA and MICB on affected cells [4]. These cells have been detected in human and mouse atherosclerotic lesions where they frequently localized to regions near necrotic cores deep within plaques and also in shoulder regions [5–11]. However, the role of NK cells in atherosclerosis is still unclear.

Early studies using beige-mutant mice on the background of low density lipoprotein (LDL) receptor (LDLR) deficiency (LDLR^−/−^) suggested a protective role for NK cells in atherosclerosis that is independent of NK cell-mediated cytolysis [7]. Subsequently, it was shown that deficiency of functional NK cells significantly reduced the size of atherosclerosis in Ly49A transgenic mice, suggesting a proatherogenic property for NK cells in atherosclerosis development [8]. These conflicting results may be partially explained by the fact that beige mutation or Ly49A transgene also influences the functions of other immune cells [11–14], making it difficult to clearly define the role of NK cells in the pathogenesis of atherosclerosis. Recently, by depleting or repleting NK cells in atherosclerosis-prone ApoE-deficient (ApoE−/−) mice, Selathurai et al. showed that NK cells promote atherosclerosis in a cytotoxicity-dependent manner [11]. Despite these studies, it remains to explore the detailed molecular mechanisms for the involvement of NK cells in atherosclerosis.

CD160 is a glycosylphosphatidylinositol (GPI)-anchored member of the immunoglobulin superfamily expressed on the majority of circulating NK cells which correspond to the nonproliferating, highly cytolytic, CD56^dim^CD16^+^ subset [15, 16]. CD160 engagement by both classical and non-classical MHC-I molecules mediates NK cell cytotoxicity and proinflammatory cytokine production of a unique profile (IFN-γ, TNF-α, and IL-6) [17–19], all of which have been implicated in atherosclerosis [20–22]. In addition, CD160 has been also described capable of binding with herpes virus entry mediator (HVEM) to augment inflammatory cytokine production and cytolytic function of NK cell [23], indicating a critical role of CD160 in regulating NK cell functions. The findings have motivated us to test the hypothesis that CD160 is involved in NK cell functioning in atherosclerosis.

## Methods

### Study subjects

This research was carried out in line with the tenets of the Declaration of Helsinki and was approved by the Institution Review Board of the Navy General Hospital of Chinese PLA. A total of 49 AS patients, including 19 patients with stable angina pectoris (SAP) and 30 with unstable angina pectoris (UAP), were recruited at Department of Cardiology of Navy General Hospital of PLA from October 2012 to June 2013. Forty-one sex and age-matched healthy subjects served as controls (Table 1). Coronary artery atherosclerosis was documented angiographically in all patients. None of the subjects had received immunomodulatory treatment during the 1-month period before sampling. Written informed consent was obtained from each participant. All samples were analyzed by flow cytometry with a Cytomics FC500 (Beckman Coulter). Sera were isolated from all study subjects and then analyzed for the concentrations of triglyceride (TG), cholesterol (Cho) and IFN-γ, TNF-α and IL-6 cytokines.

**Table 1:** Clinical characteristics of enrolled subjects

Subjects	HC (n = 41)	AS	
		SAP (n = 19)	UAP (n = 30)
Age	55.43 ± 19.54	56.46 ± 21.54	57.16 ± 18.54
Gender (M/F)	28/13	12/7	19/11
Cho (mM)	4.34 ± 1.63	4.25 ± 1.47	4.76 ± 1.51
TG (mM)	1.05 ± 0.63	1.59 ± 0.93	1.71 ± 1.03
HDL-C (mM)	–	1.29 ± 0.43	1.11 ± 0.33
LDL-C (mM)	–	2.81 ± 1.16	3.11 ± 1.23
SBP (mmHg)	116.6 ± 9.5	123.6 ± 10.5	133.6 ± 14.5
DBP (mmHg)	68.6 ± 7.7	75.6 ± 8.7	78.5 ± 9.2

Data were expressed as median ± SD.

Patients with atherosclerosis (AS); patients with stable angina pectoris (SAP); patients with unstable angina pectoris (UAP); HC (HC); cholesterol (Cho); triglyceride (TG); high density lipoprotein cholesterol (HDL-C); low density lipoprotein cholesterol (HDL-C); systolic blood pressure (SBP); diastolic blood pressure (DBP). M, male; F, female.

### Flow cytometry

Blood samples were mixed with 2 mL of ACK lysis buffer and incubated for 10 min to deplete red blood cells; after that, cells were incubated with human Fc receptor blocking buffer (eBioscience) at 4°C for 10 min followed by the incubation with specific antibodies or isotype-matched controls at 4°C for 30 min at the manufacturers’ recommended concentrations. PECY7-conjugated anti-human CD3, FITC-conjugated anti-human CD8, PECY5-conjugated anti-human CD56, PE-conjugated mouse IgM isotype control and PE-conjugated anti-human CD160 (Clone BY55; all from BD Biosciences) were used for flow cytometry analysis. Samples were washed with FACS buffer (2% BSA in PBS, 0.09% sodium azide). Pellets were resuspended in 300 mL of FACS buffer. Samples were acquired on a Cytomics FC 500 MPL (Beckmam Coulter) and analyzed by FlowJo software (TreeStar, Inc.) as reported previously [24].

### Determination of serum lipids and cytokines

Serum total Cho and TG were routinely measured in the Laboratory Department of the General Hospital of people’s Liberation Army by enzymatic methods with a commercial kit (CHOD-PAP, Boehringer-Mannheim GmbH, and Konelab TRIGLYCERIDES, Thermo Electron Co.). Serum concentrations of IFN-γ, TNF-α and IL-6 were measured by using enzyme-linked immunosorbent assay (ELISA) kits from R&D systems according to manufacturer’s instructions.

### Preparation of purified NK cells

CD3^−^CD56^+^ NK cells were obtained from PBMCs of 5 UAP patients and 5 HC using MoFlo™ XDP cell sorting system (Beckmam Coulter) as reported previously [24]. The purity of the isolated NK cells was >98% as determined by flow cytometry (Additional file 1: Figure: S1). Cells were subsequently cultured at a concentration of 2 × 10^6^ cells/ml in duplicate 24-well plates in RPMI 1640 medium (Hyclone) with 10% FBS and 100 U/mL rhIL-2 (R&D systems).

### CD160 engagement by specific monoclonal antibody (mAb)

Engagement of CD160 receptor on NK cells from AS patients or HC cultured in 24-well plates was conducted by using anti-CD160 agonistic mAb (Clone CL1-R2; MBL International) at the final concentration of 1–10 µg/ml for 16 h at 37°C in 5% CO_2_. IgG1 isotype control was also used at the same conditions. rhIL-2 (100 U/ml) was added during the incubation time. Supernatants were collected and stored at −80°C until further analysis. In some experiments, engagement of CD160 receptor on NK cells by anti-CD160 agonistic mAb were performed in the presence of 1 or 10 µg/mL control or TNF-α-neutralizing mAb (Clone 28401, mouse IgG1; from R&D systems), and then NK cells were subjected to apoptosis analysis by Annexin V/7-aminoactinomycin D staining 48, 72 and 96 h later.

### Annexin V and 7-aminoactinomycin D (7-AAD) staining

The determination of apoptotic cells was performed per manufacturer’s instruction (R&D systems). Briefly, treated cells were washed twice in cold PBS and resuspended in Annexin V-binding buffer at a concentration of 3 × 10^6^ per ml. This suspension (100 μl) was stained with 5 μl of Annexin V-FITC and 5 μl 7-AAD. The cells were gently vortexed and incubated for 15 min at room temperature in the dark. After addition of 400 μl of binding buffer to each tube, cells were analyzed by flow cytometry immediately.

### Statistics

Results were expressed as mean ± SD. All statistical analyses were performed using GraphPad Prism 5. Two-tailed unpaired student’s *t* test was used to compare the statistical difference between two groups and one-way ANOVA followed by Tukey’s multiple comparisons test was used to compare three or more groups. The Spearman correlation analysis was used to calculate the correlation coefficient. A P value <0.05 was considered as statistically significant.

## Results

### Increased CD160 expression on NK cells from AS patients

**Figure 1:**
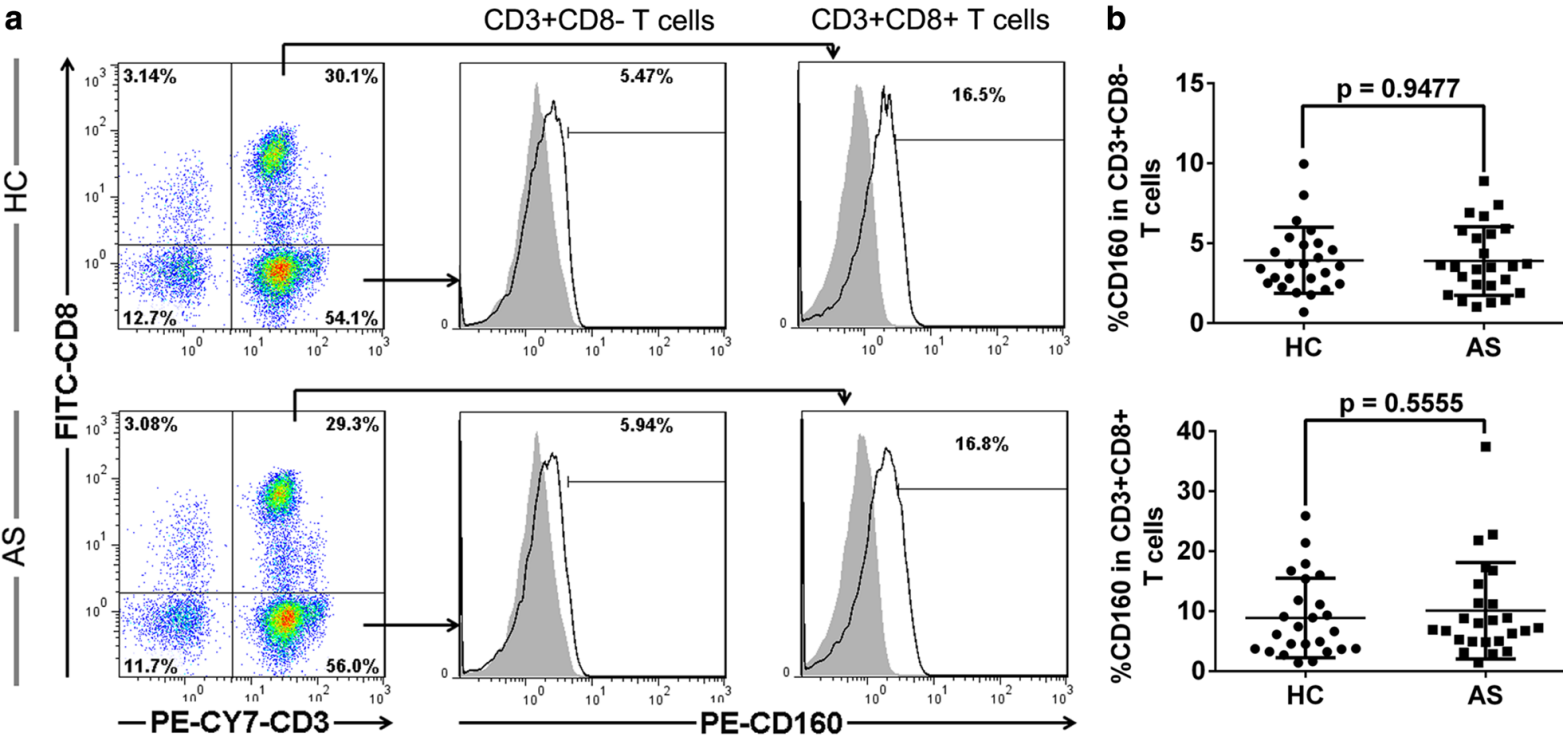
Expression levels of CD160 on CD4^+^ T and CD8^+^ T cells in AS patients. Blood samples were collected to detect CD160 expression levels on both CD3^+^CD8^+^ and CD3^+^CD8^−^ (most CD4^+^) T cells by flow cytometry. **a** Representative dot plots and histograms of CD160 expression on CD3^+^CD8^−^ (most CD4^+^) and CD3^+^CD8^+^ T cells from HC (*upper panels*) and AS patients (*bottom panels*). **b** The percentages of circulating CD160^+^CD3^+^CD8^−^ T cells (*upper graph*) and CD160^+^CD3^+^CD8^+^ T cells (*bottom graph*) in HC and AS patients with each dot representing one subject. Data are expressed as mean ± SD. Student *t* test (**b**).

**Figure 2:**
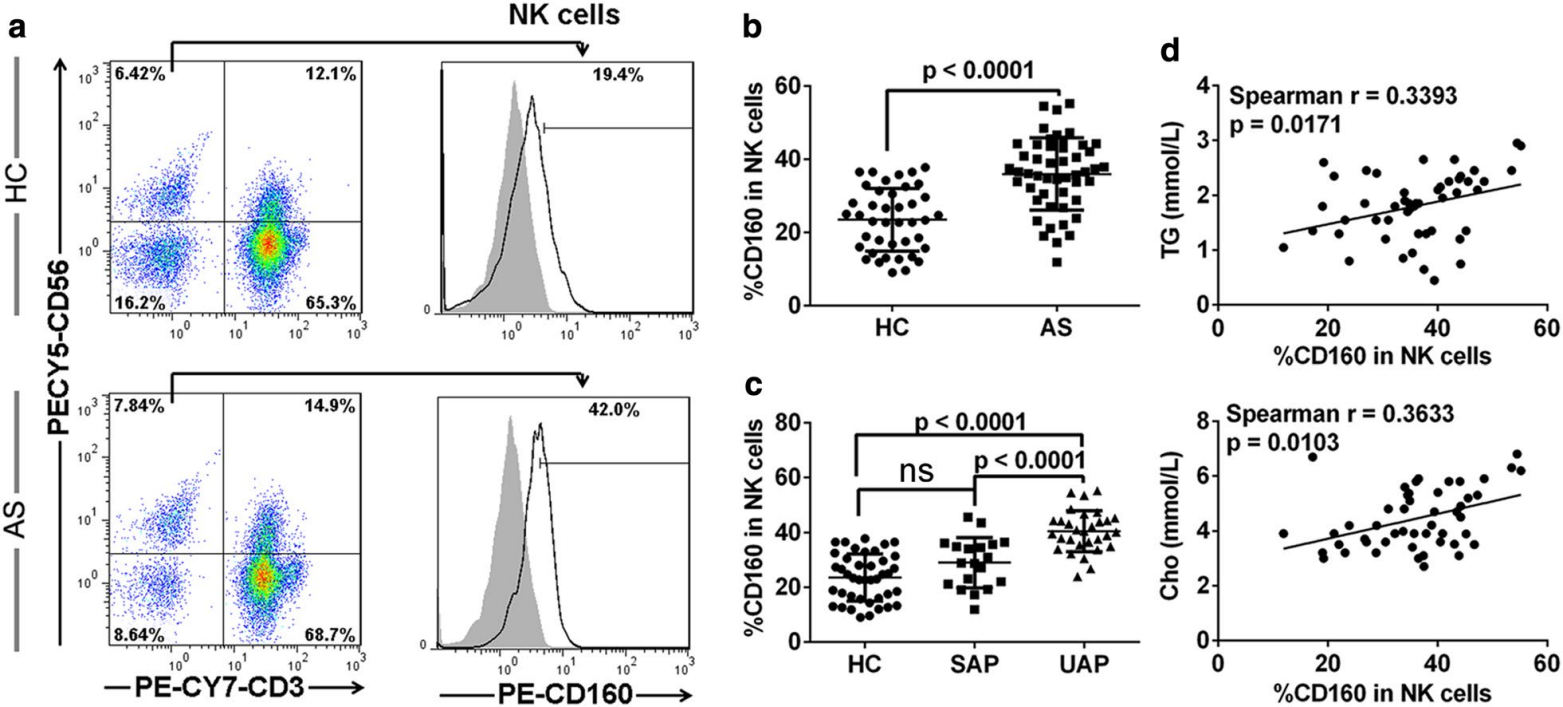
Increased expression of CD160 on NK cells in AS patients. Blood samples were collected to detect CD160 expression on NK cells by flow cytometry. **a** Representative dot plots and histograms of CD160 expression on CD3^−^CD56^+^ NK cells from HC (*upper panels*) and AS patients (*bottom panels*). **b** The percentage of circulating CD160^+^ NK cells compared between the AS patients and HC. **c** The percentage of circulating CD160^+^ NK cells compared among UAP patients, SAP patients and HC. **d** The correlation between the percentage of circulating CD160^+^ NK cells and serum TG and Cho concentrations in AS patients. Data are expressed as mean ± SD. Student *t* test (**b**), one-way ANOVA (**c**) and spearman correlation test (**d**).

To explore the potential involvement of CD160 in atherogenesis, we first compared CD160 expression levels in AS patients and the HC. Flow cytometric analysis detected a low level of CD160 expression on both CD3^+^CD8^+^ and CD3^+^CD8^−^ (most CD4^+^) T cells as previously reported [25]; however, no difference in the percentage and mean fluorescence intensity (MFI) of CD160 expression within these cells was observed between the AS patients and HC (Figure 1b; Additional file 2: Figures S2A, S1B). A representative donor analysis is shown in Figure 1a. Unexpectedly, CD160 expression (percentage and MFI) on CD3^−^CD56^+^ NK cells from patients with AS was significantly higher than that from HC (Figure 2b; Additional file 2: Figure S2C). A representative donor analysis is shown in Figure 2a. The increased CD160 expression was especially prominent on NK cells from patients with UAP (Figure 2c), suggesting a probability of CD160 expression on NK cells as a potential indicator of disease progression. Furthermore, we observed a significant correlation between the CD160 expression on NK cells and serum TG and Cho (Figure 2d), which are etiological and aggravating factors of atherogenesis [26–28].

**Figure 3:**
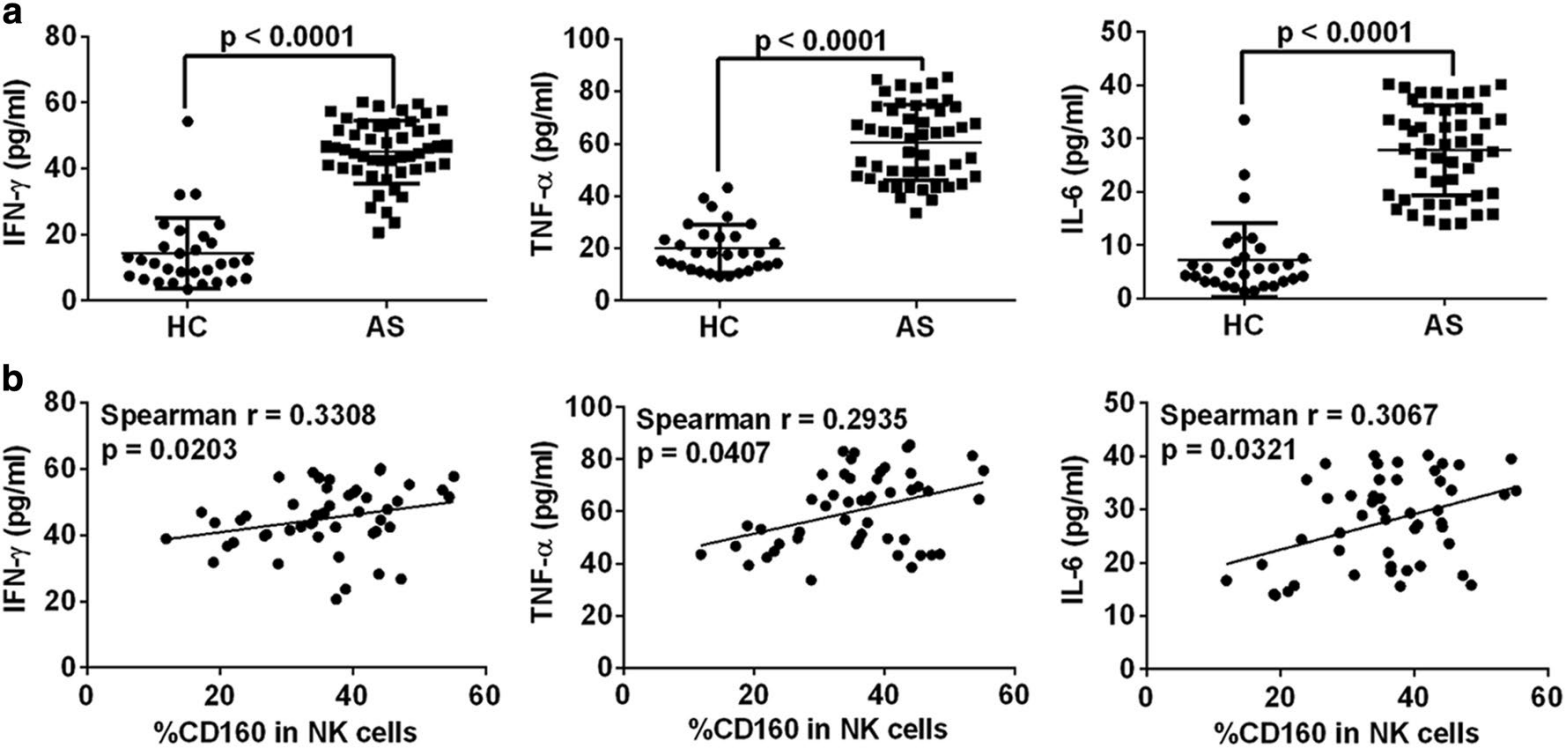
CD160 expression on circulating NK cells correlates with serum levels of inflammatory cytokines in AS patients. **a** Serum concentrations of IFN-γ, TNF-α and IL-6 determined by ELSIA. **b** The correlation analysis between the percentage of circulating CD160^+^ NK cells and serum levels of IFN-γ, TNF-α and IL-6. Data are expressed as mean ± SD. Student *t* test (**a**) and spearman correlation test (**b**).

In accordance with the nature of atherosclerosis as a chronic inflammatory disease [1, 29], the levels of plasma IFN-γ, TNF-α and IL-6, three major inflammatory biomarkers, were significantly increased in AS patients compared with those in healthy subjects (Figure 3a). Importantly, there was a positive correlation between the CD160 expression on CD3^−^CD56^+^ NK cells and the plasma level of these biomarkers in AS patients (Figure 3b). No correlation was seen in HC population (Additional file 3: Figure 3A–C). This result is also consistent with the finding that the expression of CD160 on NK cells from UAP patients was significantly higher than those in SAP patients (Figure 2c), further supporting the hypothesis that UAP patients suffer from more severe inflammation than SAP patients [19, 20]. Multivariate regression analysis further suggested a possible independent role of CD160 in predicting disease progression, but this was not statistically significant (p = 0.068), which might have been due to the small number of studied subject. In summary, our results demonstrate increased expression of CD160 on NK cells from patients with atherosclerosis, which might indicate disease progression.

### CD160 expression correlates with the reduced NK cell number among PBMCs in patients with AS

**Figure 4:**
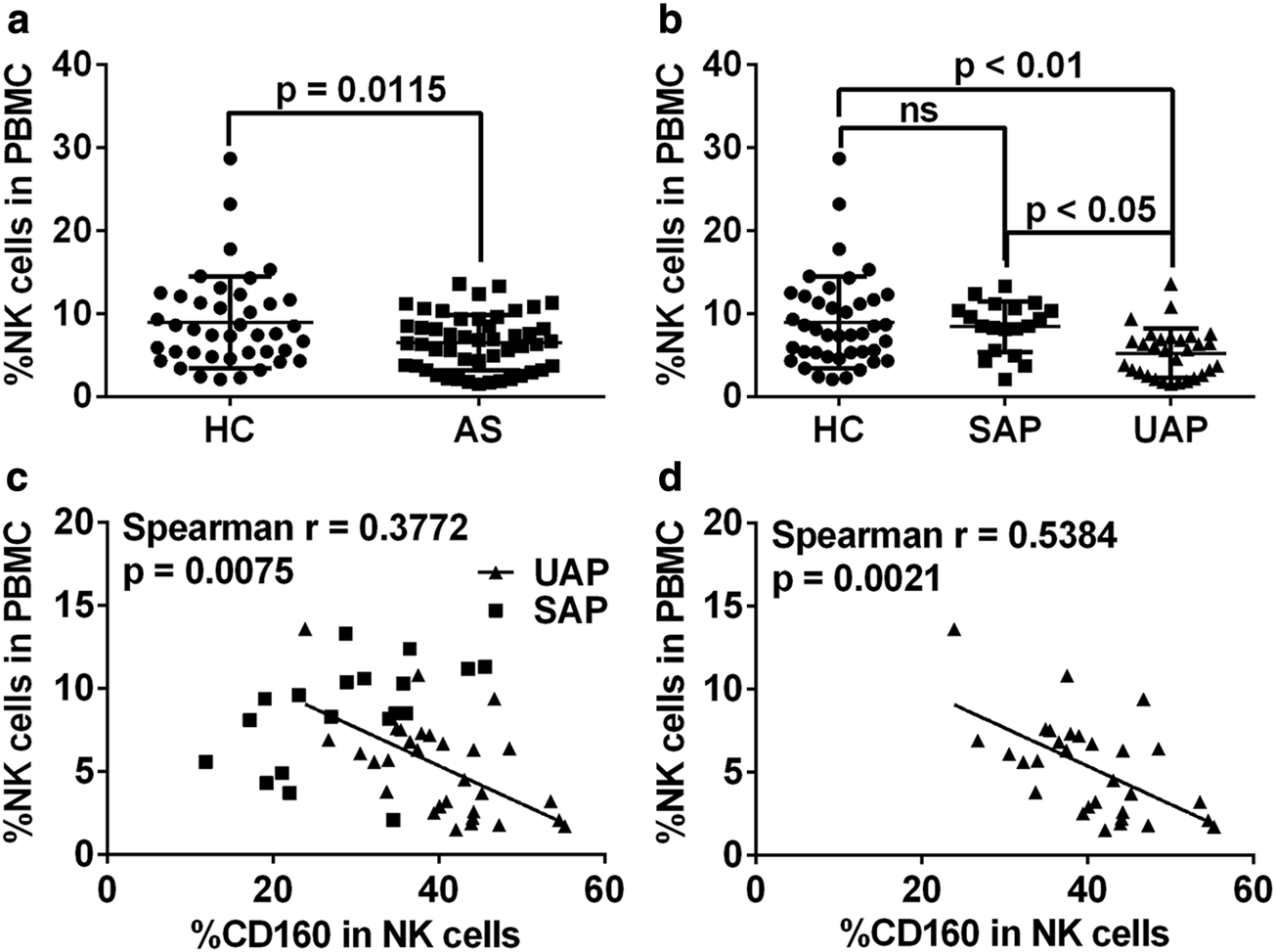
CD160 expression on NK cells is negatively correlated with peripheral NK cell number in patients with AS. **a**, **b** The percentage of NK cells among PBMCs in all AS patients (**a**) or in SAP and UAP patients (**b**). **c**, **d** The correlation between the percentage of circulating NK cells and the levels of CD160 expression on NK cells in all AS patients (**c**), including SAP (*square*) and UAP (*triangle*) patients, and in only UAP patients (**d**). Data are expressed as mean ± SD. Student *t* test (**a**), one-way ANOVA (**b**) and spearman correlation test (**c**, **d**).

As previously reported [10, 30, 31], we found reduced NK cell number in the blood from patients with AS compared with the HC (Figure 4a). Further analysis showed that the reduction of peripheral NK cell count was significantly prominent in patients with UAP than that in patients with SAP (Figure 4b). In addition, a significant inverse correlation between the level of CD160 expression and the number of NK cells was observed in all AS patients (Figure 4c) and, in particular, in the subgroup of UAP patients (Figure 4d). No correlation was seen in HC population (Additional file 3: Figure S3d).

### CD160 engagement induces TNF-α production which triggers NK cell apoptosis

**Figure 5:**
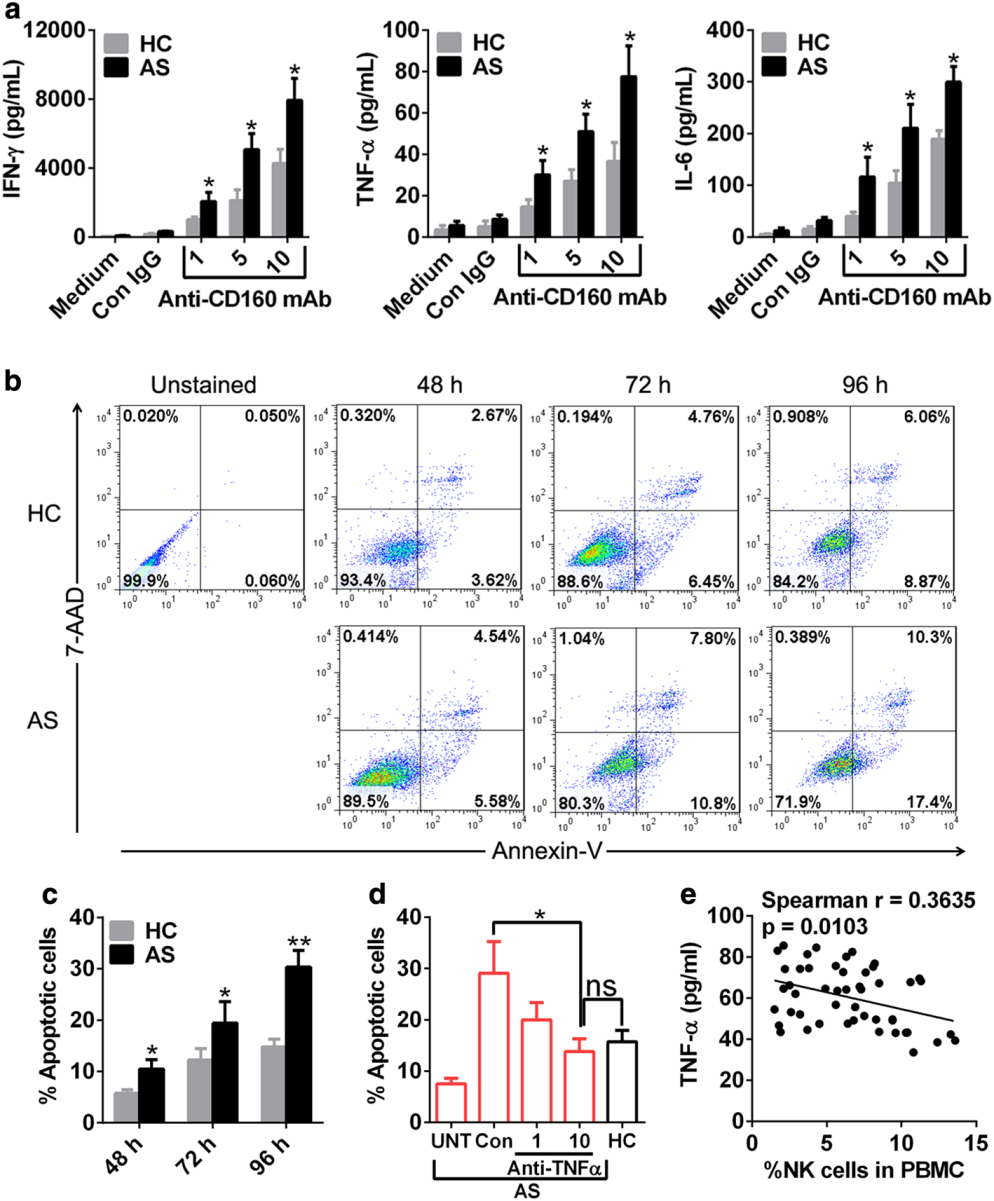
CD160 engagement triggers secretion of inflammatory cytokines and subsequent NK cell death. **a** Engagement of CD160 receptor on NK cells from AS patients (n = 5) and HC (n = 5) was conducted by using an anti-CD160 agonistic mAb at serial concentrations indicated in figures. Treatments with medium and IgG1 isotype control were used as controls. Sixteen hours later, supernatants were collected and assayed for the production of IFN-γ, TNF-α and IL-6. **b**, **c** NK cells from AS patients and HC were treated as above and their apoptosis was determined by Annexin V/7-AAD staining 48, 72 and 96 h later with apoptotic cells defined as Annexin V^+^ cells (**c**). The representative *dot plots* were shown in **b**. **d** Engagement of CD160 receptor on NK cells from AS patients were performed in the presence of control (10 µg/ml) or TNF-α-neutralizing (1 and 10 µg/ml) mAb and their apoptosis was determined by Annexin V/7-AAD staining 96 h later. Untreated NK cells from AS patients (UNT, no CD160 engagement) and treated NK cells from HC (CD160 engagement without neutralizing mAb) were used as controls. **e** The correlation of serum TNF-α with the percentage of NK cells in PBMCs with each *dot* presenting one subject. In these experiments, the purity of NK cells we used was >95% as determined by flow cytometry (Additional file 1: Figure S1). Data are expressed as mean ± SD of five AS patients and HC with each case triplicates (**a**) or representative of two independent experiments (**c**, **d**). *p < 0.05, **p < 0.01, student *t* test (**a**, **c**, **d**) and spearman correlation test (**e**).

To probe the potential role of increased CD160 expression on NK cells from patients with AS, we isolated NK cells from AS patients and HC and then subjected them to CD160 engagement by an agonistic anti-CD160 mAb. Consistent with the proinflammatory role of CD160 and its increased expression in AS patients, we detected a significantly increased production of proinflammatory cytokines, including IFN-γ, TNF-α and IL-6, from NK cells of AS patients as compared to that from HC (Figure 5a). To further explore whether CD160 engagement would lead to NK cell death, we applied AnnexinV/7-AAD double staining to dissect the apoptosis of NK cells at several time points after CD160 engagement. Notably, we observed a significantly increased apoptosis of NK cells from AS patients compared to that from HC at all evaluable time points with difference being more pronounced along time (Figure 5c). The representative dotplots were shown in Figure 5b.

As TNF-α, an important proatherogenic factor [29], has been reported to induce NK cell apoptosis [32], it is reasoned that increased TNF-α production by NK cells from AS patients after CD160 engagement may be responsible for their increased apoptosis. To test this hypothesis, we treated NK cells from AS patients with agonistic anti-CD160 mAb in the presence of control or TNF-α-neutralizing antibody to see if the increased apoptosis of NK cells from AS patients triggered by CD160 engagement could be reversed by blocking TNF-α activity. As shown in Figure 5d, blockade of TNF-α activity decreased CD160 engagement-induced apoptosis of NK cells from AS patients to the level that achieved by NK cells from HC, suggesting a critical role of TNF-α in engendering cell death of NK cells from AS patients after CD160 engagement. In addition, we found a reverse correlation between serum TNF-α concentration and circulating NK cell number, further supporting a role of TNF-α in NK cell loss in AS patients (Figure 5e).

## Discussion

Here, for the first time, we demonstrate increased CD160 expression on circulating NK cells and a critical role of CD160 in triggering inflammatory cytokine secretion and its subsequent apoptosis of NK cells during atherogenesis.

The increased CD160 expression on NK cells might be associated with an inflamed status in AS, which is supported by the following three points. First, CD160 expression on NK cells positively correlated with the levels of serum IFN-γ, TNF-α and IL-6, three important inflammation markers [29]; CD160 engagement further induces the secretion of these inflammatory cytokines, constituting a positive feedback loop to promote the inflammation. Second, CD160 expression on NK cells of UAP patients, who suffer from more severe inflammation, was significantly higher than that of SAP patients. Third, the increased CD160 expression on NK cells positively correlated with high levels of serum lipids, which can lead to higher levels of inflammation. The data suggest a close relationship between CD160 expression on NK cells and inflammation and the potential use of CD160 as an indicator for the progression of AS. Currently, the mechanisms for increased CD160 expression on NK cells in AS patients remain elusive; it is very likely that elevated CD160 on NK cells results from adaption of NK cells to the persistent inflamed microenvironment within the bodies of AS patients where multiple inflammatory mediators might induce or upregulate the transcription and/or expression of CD160 by the yet-to-identified mechanisms. Future studies are warranted to explore the detailed molecular mechanisms. In addition, CD160 transmembrane isoform (CD160-TM) has been reported to be selectively expressed by activated NK cells [33], however, the antibody we applied to detect CD160 expression cannot reliably recognize the CD160-TM [25] and the antibodies distinguishing CD160-GPI from CD160-TM are also not available, therefore, further studies are needed to concomitantly investigate the expression changes of two forms of CD160 when suitable agents are available.

Reduced NK cell number with compromised NK cell functions has been iteratively reported in AS patients [10, 30, 31], for which the mechanisms are still poorly understood. Our demonstration that engagement of CD160 on NK cells from AS patients triggered a significant production of inflammatory cytokines and subsequently NK cell death may partially explain the decreased NK cell number seen in AS patients. Previous studies have shown that combined cytokine priming, such as IL-2/IL-12 or IL-15/IL-12 stimulation, induces apoptosis of NK cells at the late stage, which can be partially prevented by TNF-α blockade [32]. Consistent with their findings, we found that neutralization of TNF-α completely prevented the increased apoptosis of NK cells from AS patients induced by CD160 engagement, suggesting a critical role of TNF-α in mediating NK cell death from CD160 engagement, which is further supported by our observation that serum concentration of TNF-α negatively correlated with quantity of NK cells in AS patients. Certainly, more in vitro functional assays with a bigger sample size are needed to exactly define the role of CD160 engagement in inflammatory cytokine production and subsequent NK cell loss.

In our study, we used an agonistic mAb to engage CD160 receptor on NK cells, however, it remains unclear the identity of molecules engaging CD160 in vivo. Previous studies showed that soluble HLA class I can induce NK cell apoptosis by the engagement of killer-activating HLA class I receptors or CD8 molecule through FasL–Fas interaction [34, 35]; as HLA class I molecules (HLA-C or HLA-G1) have been identified to be physiological ligands for CD160 [18, 36], it is likely that engagement of CD160 receptor by physiological HLA class I molecules expressed on cells reciprocally interacting with NK cells can trigger inflammatory cytokine production and subsequent apoptosis of NK cells. Further examining the localization of CD160 and its ligands by in situ immunofluorescence staining of tissue samples from AS patients would be helpful to clarify this issue.

The presence and function of NK cells in atherosclerosis development has been demonstrated both in human autopsy specimens and in various mouse models, however, the precise role and action mode of NK cells in human atherosclerosis has yet to be clarified, and the same is the exact role of CD160 on NK cells in atherosclerosis in vivo. In addition to increasing inflammatory cytokines, it is possible that elevated CD160 expression by NK cells from AS patients could increase their cytotoxic activity, contributing to increased collateral damages and associated inflammation in atherosclerotic sites since CD160 engagement by HVEM and MHC-I molecules has been described enhance the cytotoxicity of NK cells [17, 18, 23]. Due to small blood samples, we did not examine the cytotoxic activity of NK cells from AS patients, and recent study conducted in CD160-deficent mice demonstrates that CD160 controls the IFN-γ production of NK cells without affecting their cytotoxicity in tumor models [37], suggesting that CD160 on NK cells can intensify the inflammation independent of NK’s cytotoxicity. Thus, future studies are needed to figure out which effector arm derived from CD160 engagement predominately contributes to the atherogenesis.

## Conclusions

CD160 expression on NK cells from patients with AS is increased significantly, which might be associated with the inflamed microenvironment in AS. In turn, the increased CD160 expression plays a role in inflammation maintenance and NK loss by promoting inflammatory cytokine production and subsequent NK apoptosis during atherogenesis. However, the mechanisms for CD160 induction and the exact role of CD160 in atherosclerosis are not clear and need to be further investigated, especially with animal models. Our data suggest that CD160 might be used as a potential indicator for atherosclerosis progression.

## Additional files

Additional file 1:
**Figure** **S1.** The representative dot plots showing the purity of freshly isolated NK cells from a donor. The percentage of CD3-CD56 + NK cells before and after sorting was shown in each graph. Additional file 2:
**Figure** **S2.** The intensity of CD160 expression on circulating CD4^+^ cells (left), CD8^+^ cells (middle) and NK cells (right) from HC and AS patients expressed as the ratio of actual CD160 staining intensity to control staining intensity for each sample (relative MFI). Data are expressed as mean ± SD. Student *t* test (C). Additional file 3:
**Figure** **S3.** A-C. The correlation analyses between the percentage of circulating CD160^+^ NK cells and serum levels of IFN-γ, TNF-α and IL-6 in HC population. D. The correlation analyses between the percentage of circulating CD160^+^ NK cells and peripheral NK cell percentage in HC population. Spearman correlation test (A-D).

## Abbreviations

AS: atherosclerosis
LDL: low density lipoprotein
GPI: glycosylphosphatidylinositol
ELISA: enzyme-linked immunosorbent assay
MHC: major histocompatibility complex
HC: healthy controls
TG: triglyceride
Cho: cholesterol
MFI: mean fluorescence intensity
HVEM: herpes virus entry mediator
SAP: stable angina pectoris
UAP: unstable angina pectoris
RPMI: Roswell Park Memorial Institute
FBS: fetal bovine serum
FITC: fluorescein isothiocyanate
PE: phycoerythrin
7-AAD: 7-aminoactinomycin D
mAb: monoclonal antibody
TNF-α: tumor necrosis factor alpha
IFN-γ: interferon gamma
IL-6: interleukin-6
rhIL-2: recombinant human interleukin-2
SD: standard deviation
