# Supplementary figures and images for: Increased CD160 expression on circulating natural killer cells in atherogenesis
Source: J Transl Med. 2015 Jun 13;13:188. doi: 10.1186/s12967-015-0564-3 (PMC4467674; doi:10.1186/s12967-015-0564-3)

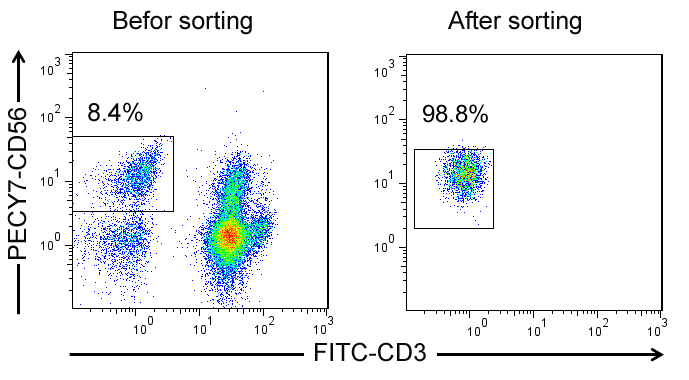

Supplement: Additional file 1: — Figure S1. The representative dot plots showing the purity of freshly isolated NK cells from a donor. The percentage of CD3-CD56 + NK cells before and after sorting was shown in each graph. [file 12967_2015_564_MOESM1_ESM.tiff]

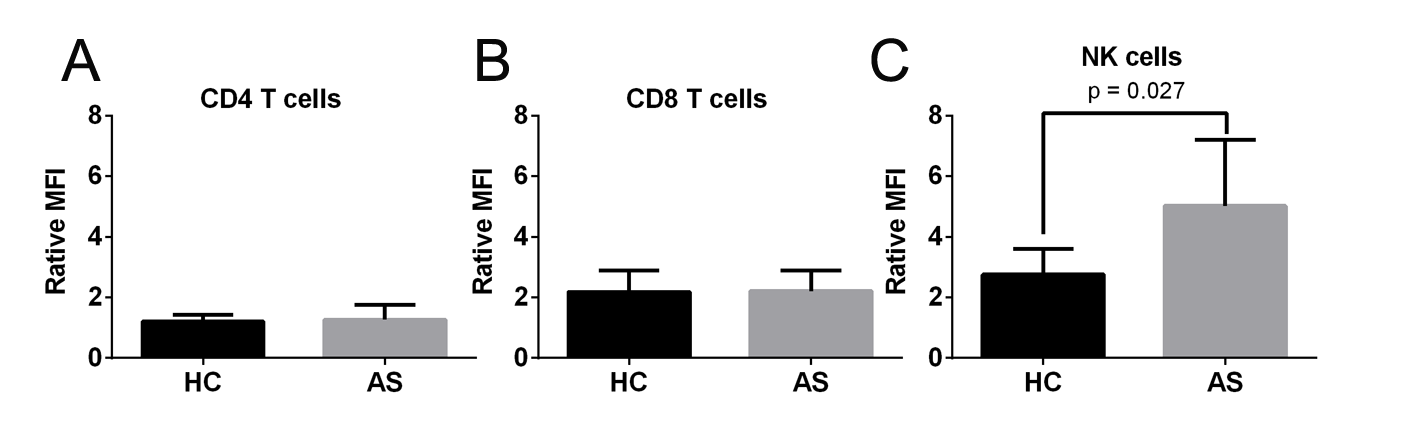

Supplement: Additional file 2: — Figure S2. The intensity of CD160 expression on circulating CD4+ cells (left), CD8+ cells (middle) and NK cells (right) from HC and AS patients expressed as the ratio of actual CD160 staining intensity to control staining intensity for each sample (relative MFI). Data are expressed as mean ± SD. Student t test (C). [file 12967_2015_564_MOESM2_ESM.tiff]

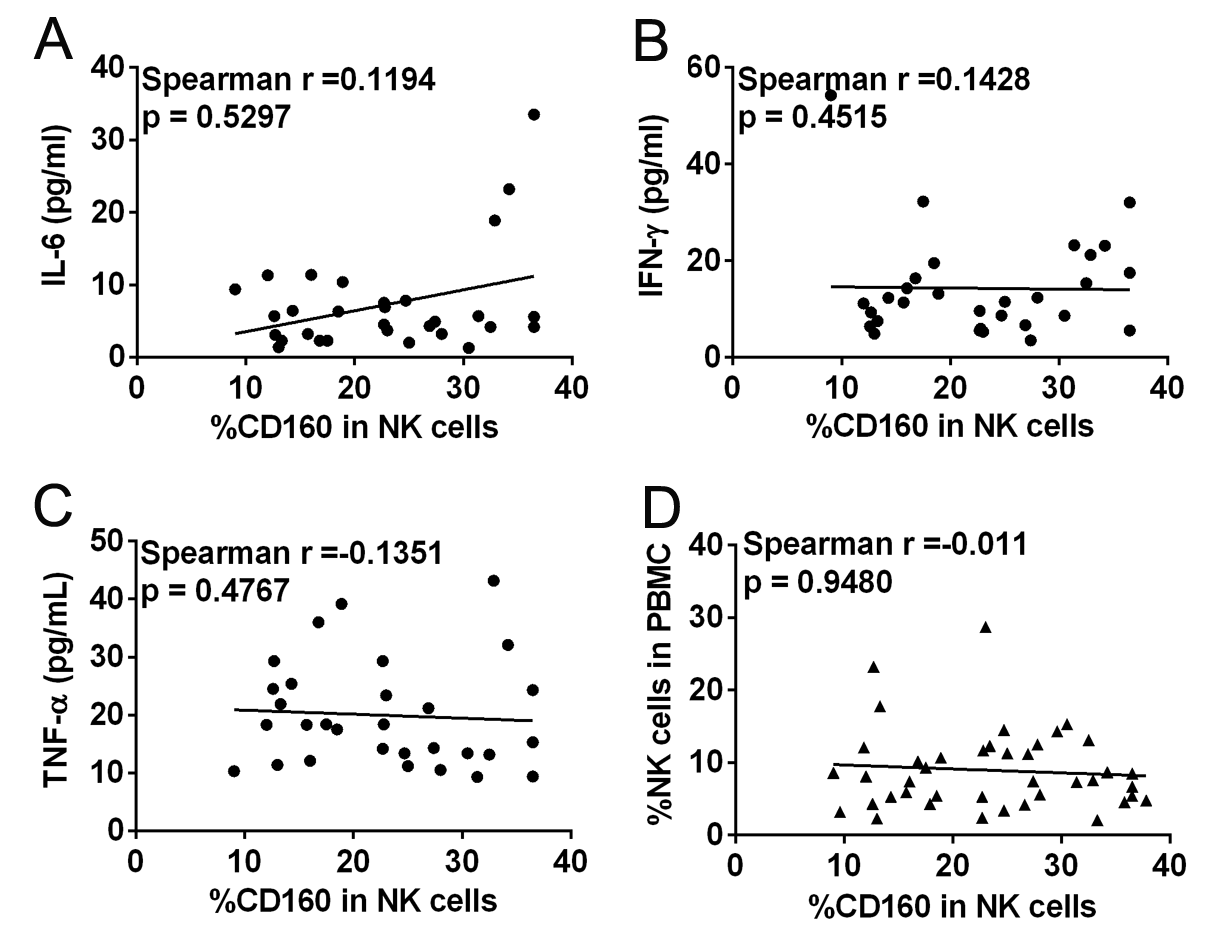

Supplement: Additional file 3: — Figure S3. A-C. The correlation analyses between the percentage of circulating CD160+ NK cells and serum levels of IFN-γ, TNF-α and IL-6 in HC population. D. The correlation analyses between the percentage of circulating CD160+ NK cells and peripheral NK cell percentage in HC population. Spearman correlation test (A-D). [file 12967_2015_564_MOESM3_ESM.tiff]
